# Repair of Thoracoabdominal Aortic Aneurysm with Thrombosed Infrarenal Component: A Modified Hybrid Technique without Aortic Cross Clamping

**DOI:** 10.1155/2017/7432032

**Published:** 2017-05-31

**Authors:** Hussam Abou-Al-Shaar, Khaled J. Zaza, Muhammad Anees Sharif, Samer Koussayer

**Affiliations:** ^1^Division of Vascular and Endovascular Surgery, Department of Surgery, King Faisal Specialist Hospital and Research Centre, Riyadh, Saudi Arabia; ^2^College of Medicine, Alfaisal University, Riyadh, Saudi Arabia

## Abstract

The authors report the successful repair of a Crawford type III thoracoabdominal aortic aneurysm (TAAA) with a thrombosed infrarenal component using a modified hybrid technique without aortic clamping in a high-risk patient. A 64-year-old male with a history of hypertension, diabetes, and severe chronic obstructive pulmonary disease presented with acute on chronic backache and bilateral short distance claudication. A computerized tomography scan demonstrated a large, nonleaking Crawford type III TAAA with thrombosed infrarenal component of the aneurysm. In addition, both common iliac arteries were occluded with the chronic thrombus. A single-stage, modified hybrid procedure involving an aortobifemoral bypass without aortic clamping, debranching of right renal, superior mesenteric, and celiac arteries as well as an endovascular repair of the thoracic aneurysm was performed. Unfortunately, despite a technically sound repair, the patient died postoperatively from a massive pulmonary embolism. TAAA with a thrombosed infrarenal aorta and bilateral common iliac arteries can be repaired using a single-stage modified hybrid procedure without aortic clamping in high-risk patients who cannot tolerate thoracotomy and aortic cross clamping.

## 1. Introduction

The natural history of thoracoabdominal aortic aneurysms (TAAA) in high-risk patients who are considered unfit for open repair is associated with high aneurysm-related mortality [1]. Improvements in anesthetic and surgical techniques permit treatment of these complex aneurysms by open surgery with improved outcomes [2, 3]. With the evolution of endovascular grafts, a hybrid technique has been viewed as a less-invasive approach for TAAA and many centers have reported good results using this technique [4, 5]. There has been a paradigm shift in the management of TAAA with the development of complex endograft technology over the last few years as good results have been reported with off-the-shelf and physician-modified fenestrated and branched devices [6, 7]. However, all approaches, whether open, hybrid, or endovascular, have their own merits and limitations. The choice of the optimal treatment for individual patients depends on the risk assessment, symptoms at presentation, aneurysm morphology, and the expertise available at the treating institution. Herein, we report the treatment of a large symptomatic TAAA in a high-risk patient. The aneurysm extended from the midthoracic aorta to the aortic bifurcations (Crawford type III) with thrombosis of the infrarenal aorta and both common iliac arteries. A single-stage modified hybrid technique without aortic cross clamping and thoracotomy was performed to treat this complex TAAA.

## 2. Case Presentation

### 2.1. History and Investigations

**Figure 1:**
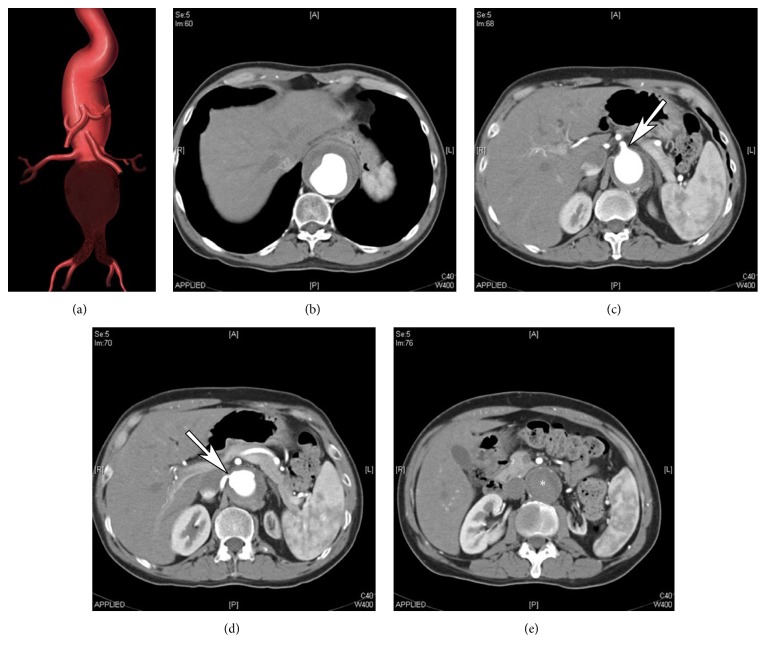
An illustration showing large type III Crawford thoracoabdominal aneurysm (TAAA) with extensive infrarenal segment thrombosis and left renal artery occlusion (a). Axial computed tomography angiogram showing the distal thoracic aorta measuring 65 mm (b), celiac artery origin (arrow in (c)), right renal artery stenosis (arrow) and left renal artery occlusion with left renal atrophy (d), and thrombosed infrarenal component of the TAAA (asterisk) measuring 42 mm (e).

A 64-year-old male presented with acute on chronic backache and short distance claudication. He had a background history of hypertension, diabetes, and severe chronic obstructive pulmonary disease (COPD). He also had a history of previous stroke with mild right hemiparesis, slurred speech, and history of epilepsy unrelated to stroke, which was well controlled with antiepileptic medications. A computerized tomography angiogram (CTA) scan demonstrated a 6.5 cm Crawford type III TAAA extending from the midthoracic aorta to the aortic bifurcation with no evidence of any leak. The celiac, superior mesenteric, and right renal arteries were patent, whereas the origin of the left renal artery was occluded with an atrophic left kidney (Figures 1(a), 1(b), 1(c), and 1(d)) and normal serum creatinine. The infrarenal component of the aneurysm and both common iliac arteries were thrombosed (Figures 1(a) and 1(e)). A plain chest radiograph revealed a left-sided pleural effusion. Doppler ultrasound of the carotid arteries showed intimal hyperplasia with few calcified plaques in the right common carotid artery but no significant stenosis.

### 2.2. Modified Hybrid Repair of TAAA

**Figure 2:**
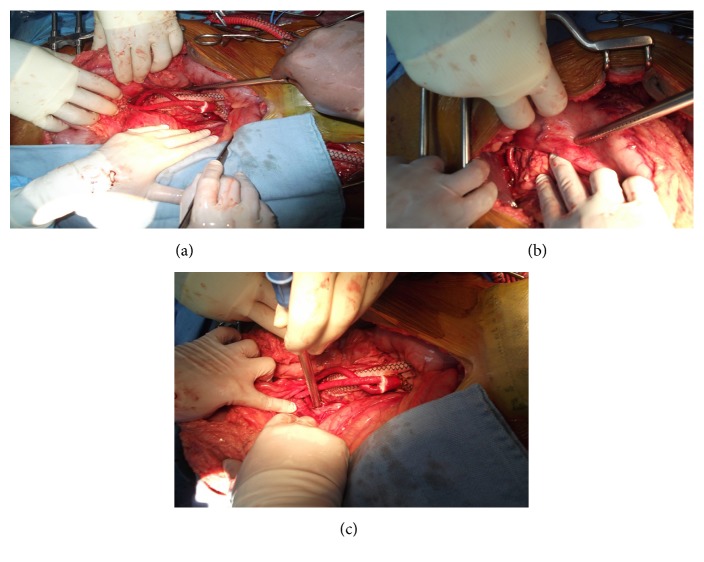
Intraoperative exposure showing the aortobifemoral, aorto-right renal, and aorto-celiac/superior mesenteric artery bypass grafts (a). Anastomosis of the graft to the right renal artery (b). Anastomosis of the graft to the superior mesenteric and celiac arteries (c).

**Figure 3:**
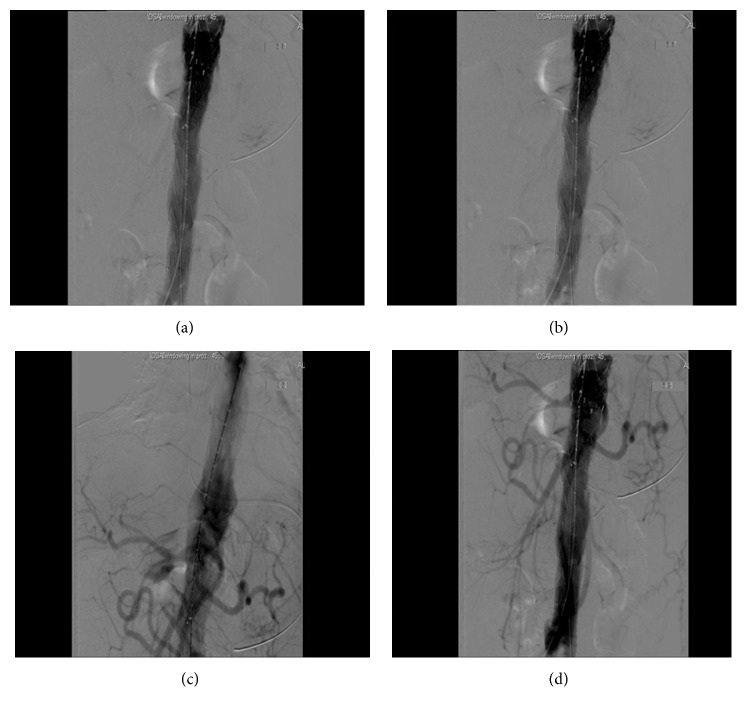
Intraoperative angiogram showing the reestablishment of the aortic blood flow through the stent graft with complete exclusion of the aneurysm (a, b, c, and d).

**Figure 4:**
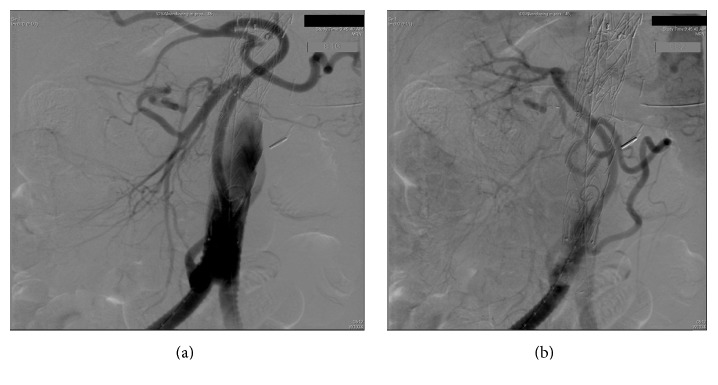
Normal blood flow in the superior mesenteric artery, celiac trunk (a), and right renal artery (b) is demonstrated on the intraoperative angiogram after retrograde graft revascularization of these vessels.

General anesthesia was induced and a spinal drainage was placed to keep the cerebrospinal fluid (CSF) pressure below 10 mmHg throughout the procedure. A midline laparotomy was performed and repair was carried out in three steps as a one-stage procedure. First, the thrombosed infrarenal component of the TAAA was exposed. In all, 5,000 international units of unfractionated heparin were administered intravenously and a 3 cm vertical incision was made at the anterior wall of the thrombosed infrarenal part of the aneurysm without aortic clamping as the aorta was thrombosed at this level with no blood flow. A 20 × 10 mm bifurcated Dacron graft was anastomosed in an end-to-side fashion to the anterior wall of the thrombosed infrarenal aorta. Once the anastomosis was completed, the thrombus in the infrarenal aorta adjacent and proximal to the aortic anastomosis was retrieved via the limbs of the bifurcated aortic graft. A pulsatile blood flow was achieved in both limbs of the graft. In the second step of the procedure, a second bifurcated graft measuring 14 × 7 mm was oriented in an upside-down direction and anastomosed to the main body of the previously sutured bifurcated graft in an end-to-side fashion for visceral perfusion. The first limb of the new graft was anastomosed to the right renal artery in an end-to-side fashion, while the second limb was sutured to the superior mesenteric artery (side-to-side) and the celiac artery (end-to-side), (Figure 2). The left renal artery was not bypassed as it was occluded with the atrophic left kidney. During the third and the final step of the hybrid procedure, two tapered Medtronic, Inc. (Minneapolis, MN), stent grafts sized 28 × 24 × 15 mm and 36 × 32 × 15 mm were deployed via the left limb of the first aortic bifurcated graft in a reversed fashion, starting from inside the bifurcated aortic graft and extending upward toward the midthoracic aorta. The two endovascular grafts covered the aorta from the midthoracic level to the main bifurcated Dacron graft with a 5 cm overlap. The endovascular grafts were balloon-molded and all debranched vessels were ligated to prevent a type II endoleak. The right and left limbs of bifurcated aortic graft were finally anastomosed to the respective common femoral arteries in an end-to-side fashion. A good pulsatile blood flow was established in both common femoral arteries and the debranching limbs of the bypass graft. Completion angiogram revealed the complete exclusion of the TAAA without any endoleak and good flow to all the debranched vessels (Figures 3 and 4). The retroperitoneum was closed covering all the grafts. The patient tolerated the procedure very well. He was hemodynamically stable during the entire procedure and was transferred to the intensive care unit in a stable condition with a spinal drain in place.

### 2.3. Postoperative Course

The patient was placed on a prophylactic dose of subcutaneous low molecular weight heparin and the CSF pressure was kept below 10 mmHg for 48 hours. He remained hemodynamically stable with no neurological deficit. Renal function deteriorated in the first 48 hours but recovered without the need for renal replacement therapy. He remained on ventilatory support in view of the severe COPD. On the fifth postoperative day, while slowly weaning off the ventilator, he became tachypneic, with signs of progressive hypoxia and right heart failure. A presumptive clinical diagnosis of pulmonary embolism (PE) was made and he was switched to intravenous heparin. While a CTA was being arranged to confirm the diagnosis of a PE, he became hemodynamically unstable with signs of worsening right heart failure and suffered cardiopulmonary arrest, which did not respond to resuscitation. The autopsy was requested but refused by the family.

## 3. Discussion

The management of TAAA is difficult and challenging. Open surgical repair as described by Crawford remained the standard surgical technique for decades [8]. The introduction of an endovascular approach in the treatment of descending aortic aneurysms by Dake et al. has demonstrated good outcomes, providing an alternative to open surgical repair [9]. However, endovascular stenting was limited by the presence of renal and visceral arteries. A combined approach consisting of an endovascular exclusion of the aneurysm with open visceral and renal graft revascularization was first described by Quinones-Baldrich in the management of TAAA [10]. The technique is based on retrograde graft revascularization of the visceral and renal arteries through laparotomy followed by endovascular stenting of the aortic aneurysmal segment.

The hybrid approach has shown many advantages over the conventional open repair in the management of TAAA. These include the avoidance of thoracotomy, aortic cross clamping, hypothermia, single lung perfusion, visceral ischemia reperfusion injury, and extracorporeal perfusion [11–14]. Thus, the hybrid procedure has been associated with a shorter postoperative recovery time, a decrease in blood loss by up to 60%, and a reduced stress response [13]. However, the risk of spinal cord ischemia, hemorrhage, endoleak, and respiratory and renal failure is still a cause for concern with the hybrid procedure [11, 12]. Paraplegia remains one of the most devastating and feared complications of hybrid procedures, but the risk is still much lower than with the open repair. The risk can be reduced by perioperative CSF drainage, as reported by many authors [15–17].

Recent studies have demonstrated a reduction in the morbidity and mortality associated with the hybrid procedure. A review of 660 patients who underwent hybrid repair for the treatment of TAAA by Canaud et al. revealed a mean perioperative mortality rate of 12.6%. The mean rate of permanent spinal cord ischemia was 3.4%, mesenteric ischemia at 4.6%, renal failure at 10.4%, severe cardiopulmonary complications at 7.8%, endovascular leak at 18%, and a mean target vessel patency rate at 94.7% during a mean follow-up of 26.2 months [12]. This rate of target vessel patency was similar to Black et al. who reported a target vessel patency of 90–95% at 36 months of follow-up [12, 14]. These results are encouraging and support the use of hybrid procedures in the treatment of TAAA as a valid and feasible alternative to open surgical repair.

The poor prognostic factors for hybrid procedure include advanced age (>60 years), cardiopulmonary comorbidity, previous cardiac surgery, diabetes, smoking, COPD, and renal insufficiency as reported by Böckler et al. [11, 18]. The patient reported in this case had multiple risk factors including diabetes, hypertension, and severe COPD. The death of this patient can be attributed to the presence of multiple risk factors superimposed by a presumptive massive PE on the 5th postoperative day. Unfortunately, there was no time for a CTA to confirm the diagnosis of PE and the offer of autopsy was turned down by the family.

Performing a hybrid repair as a single- or two-stage procedure remains controversial [12]. Some authors prefer the two-staged procedure in order to decrease the complications associated with long operative time (bleeding, ischemia, iatrogenic injury, disseminated intravascular coagulation due to hypothermia, and respiratory failure due to prolonged intubation), decrease contrast use, and ensure good spinal cord perfusion [11, 12, 15]. We, as well as other authors, believe that the single-stage hybrid procedure reduces the risk of rupture during the interval time for the second stage [14, 19]. However, according to a recent study, there has been no significant difference in clinical outcome and mortality between the two approaches [12].

In our practice, we tend to oversize the aortic stent graft by 15–20% in patients with aneurysmal disease. However, as there was a large difference in the diameter between the descending thoracic aorta (31 cm) and the bifurcated aortic graft (20 cm) in this patient, we used two tapered stent grafts which were deployed in a reverse fashion starting from inside the bifurcated aortic graft and extending upward toward the midthoracic aorta. Balloon molding of the stent graft using a compliant balloon is always performed to unfold the stent graft and strengthen the area of overlap.

The chimney technique and branched/fenestrated endovascular stent grafting for TAAA have shown promising results in many centers. Nonetheless, the medical literature lacks reported randomized clinical trials comparing them to hybrid procedures in the treatment of TAAA. The fenestrated endovascular graft is custom-made and puts high-risk patients at risk of TAAA rupture during the waiting time. However, a recent systemic review has demonstrated good results with the use of over-the-shelf and physician-modified fenestrated and branched endografts [7]. In our patient, a complex endograft or classical hybrid procedure was not feasible due to the anatomic constraints of the thrombosed infrarenal aortic aneurysm preventing endovascular access to the suprarenal component of the aneurysm. In such circumstances, the use of our modified hybrid technique is safe and feasible. As described in our technique, the bifurcated aortic graft can be sutured to the infrarenal aorta without aortic clamping and obviating the need for a thoracotomy. Adequate flow can be reestablished in the thrombosed segment of the aortic aneurysm and the limbs of the bifurcated aortic graft can be utilized as potential conduits for endovascular stenting.

In the presence of an infrarenal aortic occlusion and TAAA, the endovascular option is usually excluded from the surgical options. Most patients in this situation end up with an open repair, which carries a high risk of morbidity and mortality. Others are managed with observation alone, which has dismal outcomes in such large TAAA. We feel our technique gives the patient an alternative option to treat TAAA with less morbidity and mortality compared to an open repair. However, the procedure still carries the disadvantages of open laparotomy and general anesthesia.

This technique can be used in TAAA (Crawford type I and type II) without aortic thrombosis in patients who are at high risk for surgery and open thoracotomy. Nonetheless, an aortic clamp should be used and the aneurysm should have a narrow segment below the renal arteries to apply the aortic clamp before proceeding with the rest of the procedure.

## 4. Conclusion

Hybrid repair of TAAA is considered a potential alternative to open surgical repair, especially in high-risk patients. In the presence of an infrarenal thrombosis of the TAAA, a modified hybrid technique can be utilized by suturing a bifurcated aortic graft to the anterior wall of the thrombosed infrarenal component of the TAAA without the need for a thoracotomy and aortic cross clamping. It is followed by thrombectomy of the thrombosed aorta and debranching of the visceral arteries. Finally, the thoracic endovascular graft can be deployed via the limb of the bifurcated aortic graft to exclude the TAAA completely. We believe this technique can be considered in patients presenting with extensive infrarenal aortic thrombosis of the TAAA who are not candidates for either open or complex endovascular repair, including fenestrated, branched, or chimney techniques. Long-term results for this new technology still need to be validated.
